# Cytomegalovirus Infection Triggers the Secretion of the PPARγ Agonists 15-Hydroxyeicosatetraenoic Acid (15-HETE) and 13-Hydroxyoctadecadienoic Acid (13-HODE) in Human Cytotrophoblasts and Placental Cultures

**DOI:** 10.1371/journal.pone.0132627

**Published:** 2015-07-14

**Authors:** Kaoutar Leghmar, Nicolas Cenac, Maude Rolland, Hélène Martin, Benjamin Rauwel, Justine Bertrand-Michel, Pauline Le Faouder, Mélinda Bénard, Charlotte Casper, Christian Davrinche, Thierry Fournier, Stéphane Chavanas

**Affiliations:** 1 Centre de Physiopathologie de Toulouse Purpan, INSERM U1043, Toulouse, France; 2 CNRS U5282, Toulouse, France; 3 Université Toulouse III Paul-Sabatier, Toulouse, France; 4 MetaToul Lipidomics facility, Toulouse, France; 5 I2MC INSERM U1048, Toulouse, France; 6 Neonatal Unit, Children’s Hospital, Toulouse, France; 7 INSERM UMR-S1139, Paris, France; 8 Université Paris Descartes, Paris, France; 9 PremUP, Fondation, Paris, France; University of San Francisco, UNITED STATES

## Abstract

**Introduction:**

Congenital infection by human cytomegalovirus (HCMV) is a leading cause of congenital abnormalities of the central nervous system. Placenta infection by HCMV allows for viral spread to fetus and may result in intrauterine growth restriction, preeclampsia-like symptoms, or miscarriages. We previously reported that HCMV activates peroxisome proliferator-activated receptor gamma (PPARγ) for its own replication in cytotrophoblasts. Here, we investigated the molecular bases of PPARγ activation in infected cytotrophoblasts.

**Results:**

We show that onboarded cPLA_2_ carried by HCMV particles is required for effective PPARγ activation in infected HIPEC cytotrophoblasts, and for the resulting inhibition of cell migration. Natural PPARγ agonists are generated by PLA_2_ driven oxidization of linoleic and arachidonic acids. Therefore, using HPLC coupled with mass spectrometry, we disclosed that cellular and secreted levels of 13-hydroxyoctadecadienoic acid (13-HODE) and 15-hydroxyeicosatetraenoic acid (15-HETE) were significantly increased in and from HIPEC cytotrophoblasts at soon as 6 hours post infection. 13-HODE treatment of uninfected HIPEC recapitulated the effect of infection (PPARγ activation, migration impairment). We found that infection of histocultures of normal, first-term, human placental explants resulted in significantly increased levels of secreted 15-HETE and 13-HODE.

**Conclusion:**

Our findings reveal that 15-HETE and 13-HODE could be new pathogenic effectors of HCMV congenital infection They provide a new insight about the pathogenesis of congenital infection by HCMV.

## Introduction

Human cytomegalovirus (HCMV) is a beta herpes-virus which distribution ranges from 60 to 90% seropositive adults worldwide. HCMV infects and/or replicates in a broad spectrum of organs and cell types. Lifelong latency is established after a primary infection. Infection by HCMV is the most frequent congenital infection worldwide and a public health issue [1]. Primary infection of a seronegative mother during pregnancy, or less frequently, secondary infection in or reinfection of a seropositive mother, occurs in 0.2–2% of pregnancies and poses a 30% to 40% risk of vertical transmission to the fetus. Fetus infection may result in permanent sensorineural and neurological disabilities that affect thousands of patients each year in the USA (0.1–0.2% of all live births) [1, 2]. The most frequent permanent sequelae include mental and/or psychomotor disability, sensorineural hearing or vision loss, and/or spastic cerebral palsies, with or without orthopedic issues [3]. The direct annual costs of caring for congenitally infected patients are estimated at $1-$2 billion in the USA [2]. Congenital disease is more severe when primary maternal infection occurs in the first trimester. No vaccine is available and no reliable prognosis tool is available so far, except ultrasound examination of macroscopic brain abnormalities. Considering the dramatic health and societal cost of congenital HCMV infection, as well as the critical role of placenta in the disease, a better insight on its pathogenesis is urgently needed to provide new therapeutic and prognostic tools.

Not only placenta is the portal of entry of HCMV virions to fetus [4], it is also a genuine target of infection. Infection causes placenta dysfunction, which is thought to result in intrauterine growth restriction (IUGR), pre-eclampsia-like symptoms, miscarriages and probably stillbirth [5]. Also, placenta is an endocrine organ, which, if infected, may release deleterious soluble factors to the bloodstream or the fetus, such as inflammatory mediators or markers of infection. After infection of the uterus from ascending or hematogenous route, HCMV amplifies in the decidua, which refers to the endometrial stroma during pregnancy. Next, HCMV spreads to and replicates within the adjacent placenta in fibroblasts, fetal capillaries and two key placenta cell types, namely villous and extravillous cytotrophoblasts. The villous cytotrophoblasts (VCT) are epithelial progenitor cells, which underlie and differentiate to syncytiotrophoblasts (ST). STs populate the specialized syncytium layer surrounding the chorionic villi that float in maternal blood, and form the maternal-fetal exchange barrier. The extravillous cytotrophoblasts (EVCT) arise from the same progenitors than VCTs, invade the decidua, forming cell columns dedicated to anchor the placenta to the uterine wall, and drive vascular remodeling [6]. Infected placentas undergo villitis with lymphohistiocytic infiltrates. Placentas infected in early gestation show long-standing damage and fibrosis at the uterine-placental interface, which results in a hypoxic intrauterine environment [6]. In infected placentas from patients with IUGR, significant increase in the number of large fibrinoids with avascular villi, edema, and inflammation were observed [7].

At the molecular level, we previously showed that HCMV activates peroxisome proliferator-activated receptor gamma (PPARγ) to favor its replication in cytotrophoblasts, leading to inhibition of cytotrophoblast migration [8]. PPARγ is a ligand-activated nuclear receptor, critical in placentation and trophoblast biology [9]. Natural PPARγ ligands include 15-deoxy-∆^12,14^ prostaglandin (PG) J2 (15d-PGJ_2_), 15-hydroxyeicosatetraenoic acid (15-HETE), 9- or 13-hydroxyoctadecadienoic acid (9/13-HODE) [9] (Table 1).

**Table 1:** Name and abbreviation of each metabolite quantified in our study.

Compound	Formal name
PGD_2_	9α,15S-dihydroxy-11-oxo-prosta-5Z,13E-dien-1-oic acid
15d-PGJ_2_	11-oxo-prosta-5Z,9,12E,14E-tetraen-1-oic acid
9-HODE	(±)9-hydroxy-10E,12Z-octadecadienoic acid
13-HODE	(±)13-hydroxy-9Z,11E-octadecadienoic acid
15-HETE	15(S)-Hydroxy-5Z,8Z,11Z,13E-eicosatetraenoic acid
8-HETE	(±)8-Hydroxy-5Z,9E,11Z,14Z-eicosatetraenoic acid
12-HETE	12(S)-Hydroxy-5Z,8Z,10E,14Z-eicosatetraenoic acid

15d-PGJ_2_ arises from hydroxylation of the precursor PGD2. PGD2, 15-HETE, and 9/13-HODE are generated by oxidization of arachidonic acid (AA) or linoleic acid (LA) by cyclooxygenase (COX) or 5-/15- lipoxygenase (LOX). Our previous findings raised the possibility that activation of PPARγ in HCMV-infected trophoblast cultures could be mediated by soluble, yet unidentified, ligands [8]. Characterization of PPARγ ligands mobilized during HCMV infection is therefore critical for a better understanding of the host cell response. Here, we investigated which ligands account for PPARγ activation, both in the human cytotrophoblast line HIPEC [10] and in placental explants at early steps of HCMV infection.

## Materials and Methods

### Ethics statement

The study was performed in accordance with the French ethical guidelines and was approved by the local ethics committee of the Toulouse Children University Hospital. In this study, samples of first trimester placenta (8–11 week amenorrhea) from healthy women undergoing vaginal elective termination of pregnancy were obtained. Written informed consent was obtained from all study participants prior to sample collection. All placenta samples were anonymized before processing. Placenta samples were used only for histoculture, as described in the present article. All samples were destroyed after experimentation.

### Cells, viruses and reagents

Human immortalized extravillous cytotrophoblasts (HIPEC) were propagated in DMEM/F12 (1/1; Life technologies) containing 10% fetal calf serum (Life technologies), penicillin (100 IU/ml; Life technologies) and streptomycin (100 μg/ml; Life technologies), as described previously [10]. The human immortalized fibroblast line MRC5/CCL171 (ATCC, Manassas, VA, USA) was cultured in DMEM containing 10% bovine calf serum, penicillin and streptomycin.

We used laboratory-adapted AD169 (ATCC) or clinical VHL/E (a gift from C. Sinzger, Tubingen, Germany) HCMV strains to infect HIPEC or placenta explants, respectively. Low passage (<8 passages from the parent stock) VHL/E HCMV strains were used. For virus production, virus stocks were collected from infected MRC5 cells when cytopathic effects were >90%. Supernatants were clarified by centrifugation at 1,500 × *g* for 10 min, ultracentrifuged at 100,000 × *g* for 30 min at 4°C, resuspended in culture medium, and stored at −70°C until use. Virus titers were determined upon infection of MRC5 cells by serial dilutions of the inoculum followed by immunofluorescence analysis to count the number of nuclei immunoreactive to HCMV Immediate Early antigen (IE) 24h post infection (pi)(fluorescence unit forming assay). UV irradiation of HCMV particles was performed for 30 min in a closed propylene tube with a Spectrolyne irradiator (EF-140/F fitted with a BLE-2537S bulb, Spectronics corporation, Westbury, NY). In these conditions, HCMV virions were able to infect cells, as checked by immunostaining with an antibody specific to the tegument protein pp65 (Virusys corporation, Taneytown, MD), 30 minutes post infection; the viral genome could not be expressed, as checked by the absence of immunoreactivity to the HCMV Immediate Early antigen (IE) (Argene, Verniolle, France) 24h post infection (S1 Fig).

HIPEC were infected at a multiplicity of infection of 10 unless indicated. In these conditions, approximately 80% of cells are infected 24h post infection, as assessed by the number of cells immunoreactive to an antibody specific to HCMV Immediate Early (IE) antigen (Argene) in immunofluorescence analysis. To inactivate onboarded PLA_2_, viral suspensions were treated for one hour at room temperature by 50 μM methyl arachidonyl fluorophosphonate (MAFP; Enzo Lifesciences), ultracentifuged at 100,000 × *g* for 30 min at 4°C, washed twice with phosphate-buffered saline (PBS; Life technologies), and resuspended in culture medium. The working concentration of MAFP (50 μM) was determined by immunofluorescence analysis using HIPEC infected by HCMV particles treated by various doses of MAFP, and an antibody specific to HCMV Immediate Early (IE) antigen (Argene). Cell viability was checked by 4′6-diamino-2-phenylindole (DAPI) staining. Control viral suspensions were processed identically after incubation in the presence of the vehicle (DMSO) instead of MAFP. When HIPEC were infected with MAFP-treated virus, control uninfected HIPEC were cultured in the presence of MAFP at a concentration equivalent to that which would have been obtained without the HCMV particles washes (50 nM).

PPARγ synthetic activator was rosiglitazone (1 μM) (Sigma), and a stimulation time of 2h was used. PPARγ specific inhibitor was GW9662 (2 μM; Calbiochem), and cells were incubated with GW9662 one hour before infection. Optimal concentrations of rosiglitazone and GW9662 were determined from initial dose-effect experiments using the luciferase PPAR reporter plasmid (as detailed below). Cell viability was checked on the basis of Trypan blue exclusion, normal cell morphology, and absence of nuclei condensation or fragmentation using DAPI staining. The vehicle was assayed as a control in all experiments. Synthetic 15-HETE and 13-HODE were purchased (Sigma).

### Onboarded cPLA_2_ assay

Control, UV- irradiated, or MAFP- treated AD169 suspensions (5.10^7^ infectious particles/ml) were incubated for 1h at 37°C in the presence of the fluorescent substrate bodipy-phosphatidylcholine in PBS (100 μM; Sigma). Reaction was terminated by adding one volume of PBS/butanol-1 (v/v). Samples were then vortexed and centrifuged (5 min, 4°C at 16,000 g). The aqueous fraction was discarded, and the remaining lipids in the butanol phase were collected, dried under nitrogen, resuspended in 20 μl of chloroform-methanol-H2O (v/v/v), and loaded onto thin layer chromatography (TLC) plates. TLC was performed using chloroform-methanol-acetic acid-H2O (75/45/12/6; v/v/v/v). Fluorescence was revealed under UV light. Commercially available phospholipase A2 (lecithinase from Naja naja venom; Sigma) was used as positive control, and 0.5 U were incubated directly with bodipy-phosphatidylcholine.

### Luciferase reporter assays

We used a firefly luciferase (Luc) reporter plasmid based on a pGL4 backbone (Promega, Madison, WI, USA) and containing three PPAR responsive elements (PPREs) [8] upstream the herpes simplex thymidine kinase promoter (pGL4-PPRE-TK-luc). For normalization, we used a promoter-less renilla luciferase plasmid (pRL-null, Promega). Actively growing HIPEC were seeded in 96-well plates at a density of 25,000 cells per well. Transfection of both the reporter and normalization plasmids was performed 16 h after seeding using Genejuice transfection reagent (Novagen), according to the manufacturer's instructions. Cells were infected with HCMV, HCMV treated by MAFP beforehand, and/or stimulated with rosiglitazone (1 μM), in the presence or absence of MAFP (50 μM) or GW9662 (2 μM) in the culture medium, during the 48 h following transfection. Cell lysis was performed using Cell Culture Lysis Reagent (Promega). Luciferase activity was quantified using a Centro luminometer (Berthold).

### Oil Red O staining

HIPEC were treated by 10 μM 13-HODE or by the vehicle (DMSO), fixed in formaldehyde 4% for 30 min, washed in PBS and isopropanol 30% and then incubated for 5 min with Oil Red O solution (200mg/ml) diluted in isopropanol 30% (v/v). After washing with water, nuclei were counterstained with Harris hematoxylin (1 min), washed and observed by microscopy.

### Migration assays

For Wound healing assays, HIPEC monolayers (80% confluent) were infected or not by AD169 (MOI 3; non treated, UV- irradiated or MAFP- treated) in the presence or absence of GW9662 or rosiglitazone. In these conditions, using the live HCMV inoculum, approximately 50% of cells are infected 24h post infection, as assessed by the number of cells immunoreactive to an antibody specific to HCMV Immediate Early (IE) antigen (Argene) in immunofluorescence analysis. Cultures were scratched manually (three scratches per well) 6 h post infection, and left for 24 h at 37°C. The next day, pictures of the scratches were shot in bright field using an automated microscope (Apotome 2, Zeiss). Three fields were shot per condition. The coordinates of each field were saved by using a macro-command in the software driving the microscope (Zen, Zeiss), so that exactly the same field was shot at day 2. Next, pictures were analyzed by using ImageJ software (11). The difference between the area of the scratch in each field at days 1 and 2 was measured, and represented the surface of migration of the corresponding cell monolayer (S). Last, the index of migration (IM) was determined by normalizing S of all assays respective to the value of S for the control assay. Results are expressed as the percent variation of IM relative to the control.

Transwell migration assays were performed as described elsewhere [10] with slight modifications. HIPEC were cultured for 48 h in the presence or absence of 1 μM rosiglitazone, 2 μM GW9662, 50 nM MAFP, or infected by live, UV-irradiated, or MAFP-treated, VHL/E HCMV at a MOI of 3. The medium was renewed after 24h. After 48h, the transwell inserts were washed three times with PBS and cells were fixed for 20 min in methanol at -20°C, washed three times with PBS, and stained with DAPI. Filters were examined and photographed on a Leica DM4000B microscope. The number of nuclei detected on the filter was determined using the ImageJ software, the Fast Morphology plug-in, and a threshold size of 400 pixels square. When required, cell clusters were resolved manually using the Cell Counter plug-in. The data were normalized to the number of nuclei obtained in the control (non infected, non treated) experiment. Two independent experiments were performed. Statistical analysis was performed using the Mann-Whitney and Kruskal-Wallis tests.

### High performance liquid chromatography coupled to tandem mass spectrometry (LC-MS/MS)

LC-MS/MS was performed as detailed elsewhere [11], using HPLC grade methanol, methyl formate, and acetonitrile (Sigma–Aldrich). Deuterium-labeled lipoxin A4 (LxA4-d5), leukotriene B4 (LTB4-d4) and 5- hydroxyeicosatetraenoic acid (5-HETE-d8) (Cayman Chemicals) were mixed at a concentration of 400 ng/ml in MeOH and used as the internal standard (IS) solution. VHL/E strain was used to infect HIPEC monolayers. In all experiments, HIPEC from 10 cm² culture wells were harvested 6 h post infection in 0.2 ml of PBS, transferred to lysing matrix A (MP Biomedicals) and supplemented with 5 μl of IS solution.

Cells were lysed using a spin homogeneizer (Fastprep, MP Biomedicals) with 2 cycles of 20 sec at 5 000 rpm. 10 μl of the lysed cell suspension were added to 200 μl of 0.1 M NaOH for subsequent protein quantification using a Bradford assay (BioRad). The remaining of the lysate was supplemented with 200 μl methanol, vigorously shaken, and centrifuged for 15 min at 1 000 x g at 4°C. The supernatants were collected and stored at -80°C until lipid extraction. Lipid amounts from cell lysates were expressed in pg per mg of protein in the lysate. HIPEC culture supernatants were collected 6 hours post infection, supplemented with 300 μl of ice cold methanol and 5 μL of IS solution, clarified by a centrifugation at 1000g for 15 minutes, and stored at -80°C until lipid extraction. Lipid amounts from supernatants were expressed as pg/ml.

Lipid preparation from all samples was carried out through solid-phase extraction using hydrophobic polystyrene-divinylbenzene resin in dedicated 96-well plates (Chromabond multi96 HR-X 50 mg; Macherey-Nagel). After conditioning of the plate with methanol and sample loading, the plates were washed twice with H_2_O/MeOH (90/10, v/v) and dried under aspiration for 15 min. Lipids were eluted with methanol (2 ml), dried under nitrogen, dissolved again in methanol (10 μl) and transferred to liquid chromatography tubes before LC–MS/MS analysis.

LC-MS/MS analysis was performed using an UHPLC system (LC1290 Infinity, Agilent) coupled to a 6460 triple quadrupole mass spectrophotometer (Agilent Technologies) fitted with electro-spray ionization interface. Separation was done at 40°C on a Zorbax SB-C18 column (2.1 mm–50 mm–1.8 μm) (Agilent Technologies). The compositions of mobile phase A and B were water, acetonitrile (ACN) and formic acid (FA) (75/25/0.1) and ACN, FA (100/0.1), respectively. Compounds were separated with a linear gradient from 0 to 85% B in 8.5 min and then to 100% B at 9 min. Isocratic elution continued for 1 min at 100% B, then 100% A was reached at 10.2 min and maintained to 11 min. The flow rate was 0.35 ml/min. The autosampler was set at 5°C and the injection volume was 5 μL. Source conditions were as follows: negative ESI mode; source temperature = 325°C, nebulizer gas (nitrogen) flow rate = 10 L/min, sheath gas (nitrogen) flow rate = 12 L/min, sheath gas temperature = 400°C and spray voltage = −3500 V. Data were acquired in MRM mode. For each compound the best conditions of separation and quantification were defined: retention time in minute (RT), specific Q1/Q3 transition (T) fragmentor (F) and collision energy (CE). Peak detection, integration and quantitative analysis were performed using Mass Hunter Quantitative analysis software (Agilent Technologies). At least three independent experiments were performed, each in triplicate wells.

### Placenta histoculture assays

Twelve first trimester HCMV-negative placentas (8–11 weeks amenorrhea) were obtained following elective abortions carried out by vacuum aspiration at the Paule de Viguier Hospital (Toulouse, France). Fresh placentas were processed as described elsewhere [12]. We used method B according to Lopez et al [12]. Briefly, 3 x 10^5^ MRC5 cells were seeded in 10 cm² wells 5 days before placenta collection, and were infected by HCMV (VHL/E strain) at a MOI of 3 the day after. Collected placenta was washed with DMEM supplemented with 15% FCS, 1% penicillin-streptomycin, 0.1% gentamicin, 1% amphotericin B, and cut to obtain several villi blocks of approximately 3 mm². Blocks were placed onto sterile gelatin sponge substrates (Gelfoam) at the air-liquid interface, in 10 cm² culture wells containing infected MRC5 cells or uninfected MRC5 for the control (day 0). There was no direct contact between the MRC5 cells and the placental blocks. At day 5 post infection, the placental explants were placed into new 10 cm² wells without supporting MRC5 layer, washed three times with PBS, and left in fresh culture medium. The culture media were collected at days 6 and 12 post infection and stored at -80°C until lipid analysis

### Statistical analysis

Statistical analyses were performed with the StatEL plugin (Adscience) for Excel (Microsoft) using Kruskal-Wallis test, except for data from placenta histoculture assays, which were analyzed with the Prism program (Graphpad) using the Wilcoxon test. Error bars are SEM. p values <0.05 or less were considered significant.

## Results

### HCMV particles carry onboarded cPLA_2_ activity

**Fig 1:**
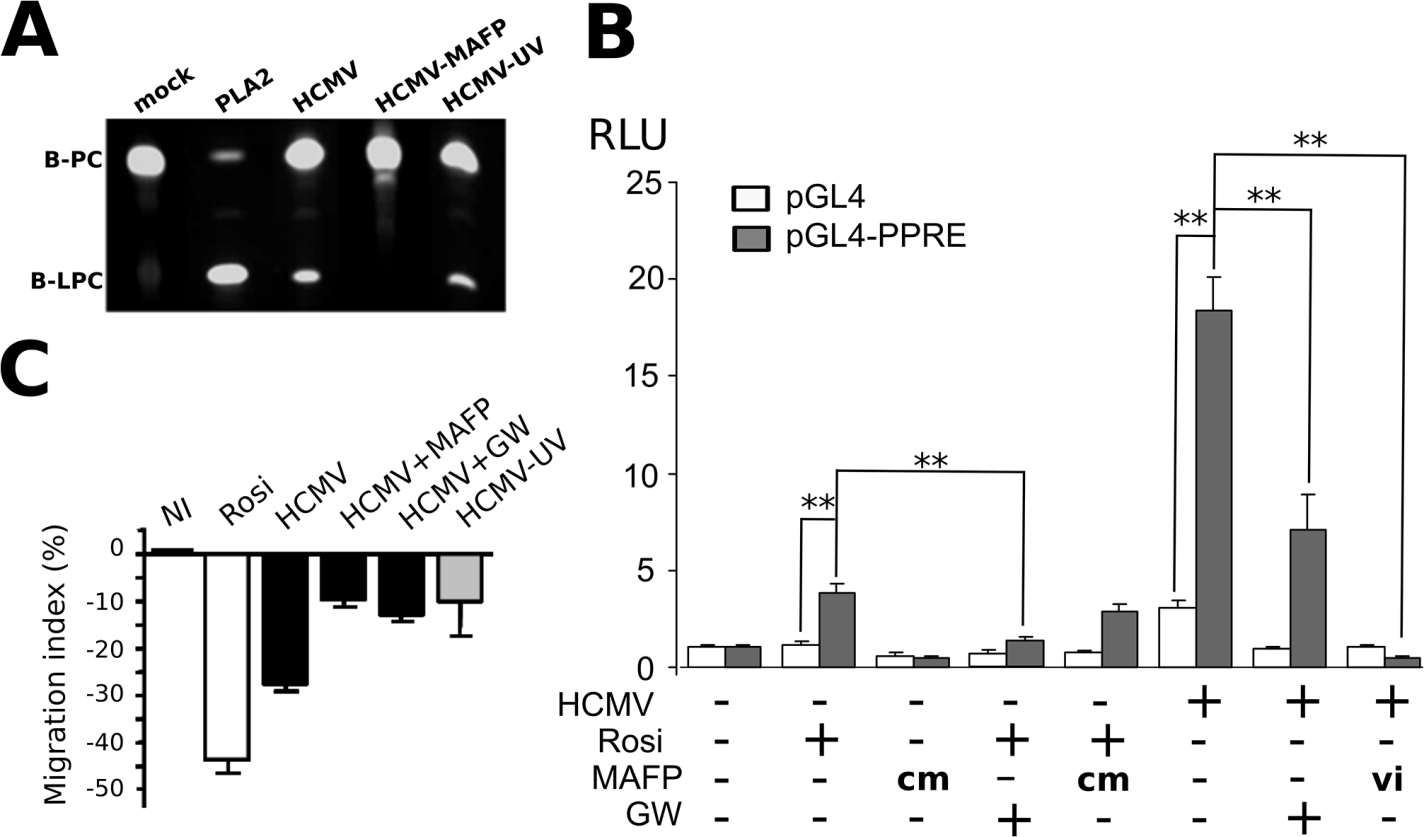
Onboarded cPLA_2_ is required for PPARγ activation and inhibition of cytotrophoblast migration. (A) TLC analysis of bodipy-phosphatidylcholine (B-PC) incubated in the presence of buffer (mock), purified PLA_2_ (PLA2), native HCMV particles (HCMV), HCMV particles pre-treated by MAFP (HCMV+MAFP) or UV-irradiated (HCMV-UV). (B) PPARγ activity luciferase assay performed with HIPEC in various conditions, using a reporter plasmid responsive to PPARγ (pGL4-PPRE) or a control plasmid (PGL4), 48 h pi or post treatment onset. HCMV: HIPEC infected by live HCMV particles; vi: the viral inoculum was treated beforehand by MAFP; cm: control with 50 nM MAFP in the culture medium; GW: PPARγ inhibitor GW9662; Rosi: PPARγ agonist rosiglitazone. RLU: relative luciferase units; **: p < 0.01 (Kruskal-Wallis test). The assay was repeated twice.(C) Wound-healing assays. HCMV+MAFP: the viral inoculum was treated beforehand by MAFP; HCMV+GW: the HIPEC were infected in the presence of GW9662; HCMV-UV: the viral inoculum was UV-irradiated. Results are expressed as the percent variation relative to the control. The figure shows results of a representative experiment, out of two independent experiments, each comprising triplicate measures. NI: non-infected.

We first investigated the biochemical bases of the generation of PPARγ agonists derived from polyunsaturated fatty acids (PUFA) in infected HIPEC. Release of arachidonic acid (AA) and linoleic acid (LA) from membrane glycerophospholipids is catalytically driven by calcium-dependent phospholipase A_2_ (cPLA_2_) [13]. It has been demonstrated that HCMV particles carry an onboarded, host cell-derived cPLA2 activity (oPLA2), required for virus replication [14]. We hypothesized that oPLA2 delivered to the infected cell could contribute to the biosynthesis of PPARγ activators. Since UV-irradiated HCMV does not activate PPARγ in HIPEC [8], we first investigated whether UV irradiation abolished o PLA_2_ activity in HCMV particles (Fig 1A). To determine whether HCMV virions carried active oPLA_2_, we examined the hydrolysis of a fluorescent bodipy-phosphatidylcholine substrate (B-PC) to bodipy-lysophosphatidylcholine (B-LPC) when incubated in the presence of HCMV particles. In this assay, generation of B-LPC thus indicates PLA_2_ activity. Thin layer chromatography showed B-LPC generation from B-PC incubated with native HCMV particles (Fig 1A). PLA_2_ activity was detected from B-PC incubated with UV-irradiated HCMV particles, indicating that UV irradiation did not inactivate this oPLA_2_ (Fig 1A). In contrast, HCMV particles treated beforehand by the specific PLA_2_ inhibitor methyl arachidonyl fluorophosphonate (MAFP) showed efficient inhibition of oPLA_2_ (Fig 1A). These data show that HCMV particles actually carry onboarded cPLA_2_, which is sensitive to inhibition by MAFP, but not to UV irradiation. Therefore, we used MAFP to inactivate o PLA_2_ in subsequent experiments.

### Onboarded cPLA_2_ activity is required for effective PPARγ activation and for inhibition of HIPEC migration

We had previously shown that only transcriptionally competent HCMV particles were able to cause PPARγ activation in HIPEC [8]. It was however unknown whether lipid mediators generated from LA or AA through o PLA_2_ activity actually contribute to PPARγ activation. Hence, we investigated whether MAFP-treated HCMV particles were able to activate PPARγ in HIPEC by using a PPARγ -reporter luciferase assay (Fig 1B). As expected, infection by native HCMV particles induced a dramatic and significant (p<0.01) increase in PPARγ activity in HIPEC (Fig 1B). Likewise, stimulation of uninfected HIPEC by the selective PPARγ ligand rosiglitazone [15] also resulted in significant activation (Fig 1B). PPARγ activation was strongly and significantly inhibited when HIPEC were cultured in the presence of the PPARγ selective ligand GW9662 [16], even if stimulated by rosiglitazone or infected by live HCMV (Fig 1B). We also investigated the effect of MAFP treatment of HCMV particles upon PPARγ activation in HIPEC. In our system, it is very unlikely that HIPEC had been in contact with active doses of MAFP, since viral particles were carefully washed by ultracentrifugation before being used to infect HIPEC. In any event, to rule out any effect from possible contaminant MAFP in the inoculum, we performed control assays with uninfected HIPEC in the presence of MAFP at a concentration equivalent to that would have been obtained without the HCMV particles wash, i.e., 50 nM. We found no detectable effect of MAFP on PPARγ activity in these control assays (Fig 1B). In contrast, we observed that infection of cytotrophoblasts with MAFP-treated HCMV particles abolished PPARγ activation associated with infection (Fig 1B). These findings suggested that oPLA_2_ activity from HCLMV particles is required for effective PPARγ activation in HIPEC.

We further explored the impact of oPLA_2_ on infected HIPEC. We previously reported that PPARγ activation in HIPEC impairs cell migration, should PPARγ be activated by rosiglitazone stimulation or HCMV infection [8]. Therefore, we investigated whether MAFP-treated HCMV particles altered HIPEC migration abilities by using wound-healing assays (Fig 1C). Infection of cytotrophoblasts with MAFP-treated HCMV particles decreased PPARγ-dependent inhibition of cell migration in a manner similar to that caused by UV irradiation of the inoculum, or by treatment of HIPEC with the PPARγ synthetic inhibitor, GW9662 (Fig 1C). Altogether, these results suggest that oPLA_2_ is required for PPARγ activation and inhibition of cytotrophoblast migration.

### 15-HETE and 13-HODE are the predominant PUFA-derived agonists of PPARγ in infected HIPEC

**Fig 2:**
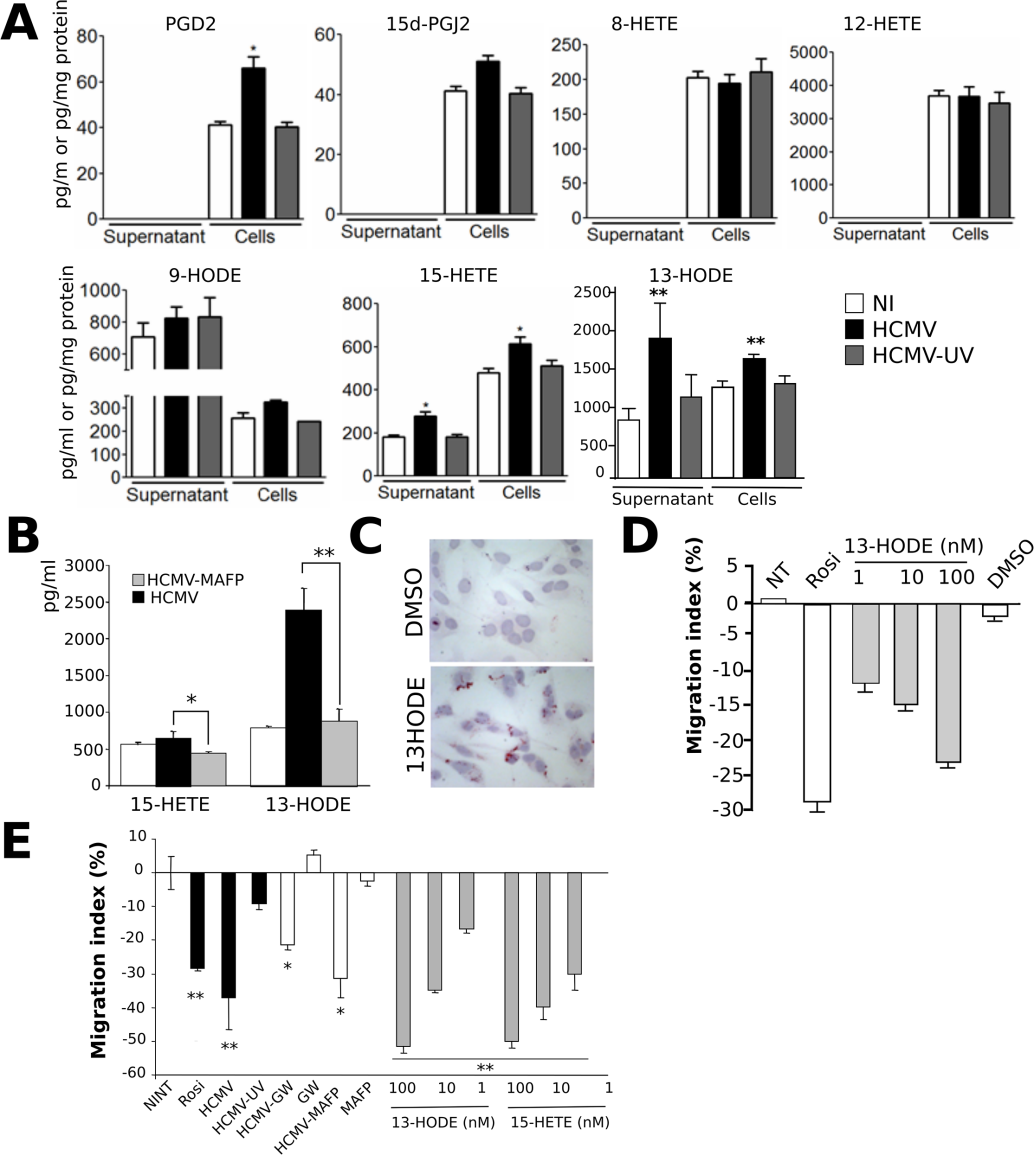
15-HETE and 13-HODE are released by HIPEC infected by live HCMV. (A) LC-MS/MS analysis of the PUFA-derived lipids in HIPEC uninfected (NI), infected with live HCMV (HCMV) or UV-irradiated HCMV (UV-HCMV) (expressed as pg/mg of protein) and from the corresponding culture supernatants (expressed as pg/ml). (B) LC-MS/MS analysis of 13-HODE and 15-HETE in the culture medium of HIPEC uninfected (NI), infected by live HCMV (HCMV) or by HCMV treated by MAFP (HCMV-MAFP). (C) Red Oil O staining of uninfected HIPECs stimulated by 13-HODE or treated by the vehicle (DMSO). (D) Wound healing assay using uninfected HIPEC treated by different doses of 13-HODE, rosiglitazone (Rosi), or the vehicle (DMSO), or untreated (NT). The results shown are from one representative experiment, out of two independent experiments, each comprising triplicate measures. (E) Transwel migration assay using uninfected HIPEC or HIPEC infected by live HCMV (HCMV), UV-irradiated HCMV (HCMV-UV) or HCMV treated by MAFP (HCMV-MAFP) (MOI = 3), in the presence or the absence of 1μM rosiglitazone, 2 μM GW9662 (GW), 50 nM MAFP (MAFP), or 13-HODE (13-HODE) or 15-HETE (15-HETE) at various concentrations (nM). Shown are the results of two independent experiments. *: p < 0.05; **: p < 0.01; ***: p < 0.001.

We next hypothesized that o PLA_2_ activity contributes to the biosynthesis of PPARγ ligands in infected HIPEC. This prompted us to search for PPARγ activating lipids generated in HIPEC at early stages of infection. To identify putative PPARγ activators generated among AA or LA metabolites, we used a novel, rapid and sensitive method based on high performance liquid chromatography coupled to tandem mass spectrometry (LC-MS/MS) [11] using lysates and conditioned culture media collected at 6 hours pi (Fig 2A). Candidate PPARγ agonists were 9/13-HODE, 15-HETE, and 15d-PGJ_2_. We also investigated the amounts of other PUFA-derived lipids: 8/12-HETE, which have not been identified as PPARγ agonists to date, and PGD2, because it is the precursor of 15d-PGJ_2_. The optimal conditions determined for lipid separation for each compound are provided (Table 2).

**Table 2:** Optimal conditions for lipid separation by LC-MS/MS.

Compounds	Q1/Q3 transition	Fragmentor (V)	Collision energy (V)
LxA4-d5*	356/115	100	6
PGD_2_	351/271	100	12
15d-PGJ_2_	315/271	90	2
LTB4-d4*	339/197	120	6
13-HODE	295/195	140	8
9-HODE	295/171	135	8
15-HETE	319/219	110	2
12-HETE	319/208	110	2
8-HETE	319/155	110	4
5-HETE-d8*	327/155	100	2

* Deuterated compound used as an internal standard.

For each metabolite, we also determined retention time, line equation, coefficient of determination, maximum percent residual, limit of detection and quantification (Table 3).

**Table 3:** Retention time (RT), line equation, coefficient of determination (r2), maximum percent residual, limit of detection (LOD), limit of quantification (LOQ) and attributed internal standard (IS) for each metabolite.

Compound name	RT min	Equation	r²	Max % Residual	LOD ng/mL	LOQ ng/mL	IS
PGD_2_	3.87	y = 1.767076 * x + 1.199E-005	0.9996	1.872	0.24	0.49	LxA4-d5
15d-PGJ_2_	7.60	y = 4.119598 * x—5.150E-004	0.9978	10.177	0.24	0.49	LTB4-d4
9-HODE	7.99	y = 0.451368 * x + 8.281E-004	0.9997	3.420	0.12	0.24	5HETE-d8
13-HODE	7.96	y = 0.550079 * x + 0.0017	0.9966	3.980	0.48	0.98	5HETE-d8
15-HETE	8.19	y = 0.438428 * x—2.109E-005	0.9970	5.427	0.48	0.98	5HETE-d8
8-HETE	8.48	y = 0.322877 * x + 2.779E-005	0.9992	2.816	0.48	0.98	5HETE-d8
12-HETE	8.50	y = 0.095890 * x + 9.751E-005	0.9974	3.450	1.90	3.60	5HETE-d8

LC-MS/MS analysis revealed a wide range of PUFA-derived lipids in HIPEC or in conditioned culture media (Fig 2A). No PGD2, 15d-PGJ2, or 8/12-HETE could be detected from the culture supernatants of infected or control HIPEC (Fig 2A). There was no change in the amounts of 15d-PGJ2 or 8 /12-HETE in infected HIPEC as compared to the uninfected controls (Fig 2A). Likewise, no significant change was observed in levels of cellular or secreted 9-HODE in infected HIPEC as compared to the controls (Fig 2A). In contrast, we found moderately but significantly increased levels (approximately 1.5 fold) of 15-HETE in lysates (p< 0.02) or supernatants (p< 0.029) from HCMV-infected HIPEC as compared to the uninfected controls (Fig 2A). 15-HETE amounts in uninfected control cell lysates were detected at 459 pg (1.4 pmol) per mg of protein and rose to 659 pg (2 pmol) per mg of protein in infected HIPEC. In the conditioned culture supernatants, these amounts were measured at 164 pg/ml (0.5 nM) in uninfected cultures, compared to 316 pg/ml (0.9 nM) in infected cultures. In addition, LC-MS/MS analysis revealed a strong (> 2.4 fold) and significant (p< 0.021) increase in the mean amount of 13-HODE secreted from HCMV-infected HIPEC (Fig 2A). Indeed, amounts of 13-HODE were far greater than that of 15-HETE. Secreted 13-HODE amounts in supernatants ranged from 282 pg/ml (0.9 nM) in uninfected HIPEC cultures to 3,056 pg/ml (9.8 nM) in infected HIPEC cultures. We also observed a significant, albeit moderate (>1.4 fold, p< 0.026), increase in the mean amount of 13-HODE in infected HIPEC lysates (Fig 2A). Cytoplasmic 13-HODE amounts ranged from 1,091 pg/mg of protein (3.7 nM) in uninfected HIPEC to 1,674 pg/mg of protein (5.6 nM) from infected HIPEC (Fig 2A). Amounts of 13-HODE and 15-HETE were not significantly different from uninfected controls when the inoculum was irradiated by UV light (Fig 2A). In contrast, infection by HCMV particles treated beforehand by MAFP abolished the increase in the quantity of secreted 13-HODE and 15-HETE 6 h pi (Fig 2B), indicating that active oPLA_2_ is needed for their efficient biosynthesis.

13-HODE and 15-HETE have been well characterized as *bona fide* PPARγ agonists [9, 17]. We nevertheless verified the ability of such PUFA-derived agonists to activate PPARγ in HIPEC, using 13-HODE as a model. Treatment of uninfected HIPEC by 10 nM 13-HODE induced accumulation of lipid droplets, indicative of active PPARγ (Fig 2C). We also checked whether 13-HODE and/or 15-HETE treatment altered the migratory properties of HIPEC. Preliminary wound healing assays showed that treatment of uninfected HIPEC by 13-HODE reduced cell migration in a dose-dependent manner, recapitulating the effect of infection, similarly to rosiglitazone stimulation (Fig 2D). Because HIPEC retain the invasive properties of primary EVCT [10], we next investigated the outcomes of 13-HODE or 15-HETE treatment on HIPEC migration capacities using transwell migration assays. As expected, a significant decrease in migration was found in infected HIPEC (p<0.0015) or uninfected HIPEC stimulated by rosiglitazone (p<0.0065) as compared to the uninfected, untreated controls (Fig 2E). This inhibition of migration was strongly attenuated when HIPEC were infected by live HCMV in the presence of the PPARγ inhibitor GW9662 (Fig 2E). Treatment of HIPEC by GW9662 or MAFP, or culture of HIPEC in the presence of UV irradiated HCMV did not result in any significant change in migration as compared to the control (Fig 2E). GW9662 treatment caused a slight, albeit non significant (p<0.73), increase in migration, probably through inhibition of basal PPARγ activity in HIPEC. Noteworthy, incubation of HIPEC in the presence of MAFP-treated HCMV showed a significant decrease in migration as compared to the uninfected control (p<0.034) (Fig 2E), possibly because of endogeneous PLA_2_ activity substituting for that of oPLA_2_ within the 48 hours of incubation. Lastly, stimulation by 13-HODE or 15-HETE resulted in significant, dose-dependent, impairment of migration as compared to the unstimulated controls (p<0.004), what confirmed that 13-HODE and 15-HETE showed anti-migratory properties upon HIPEC.

### Placenta histocultures secrete increased amounts of 15-HETE and 13-HODE when infected by HCMV

**Fig 3:**
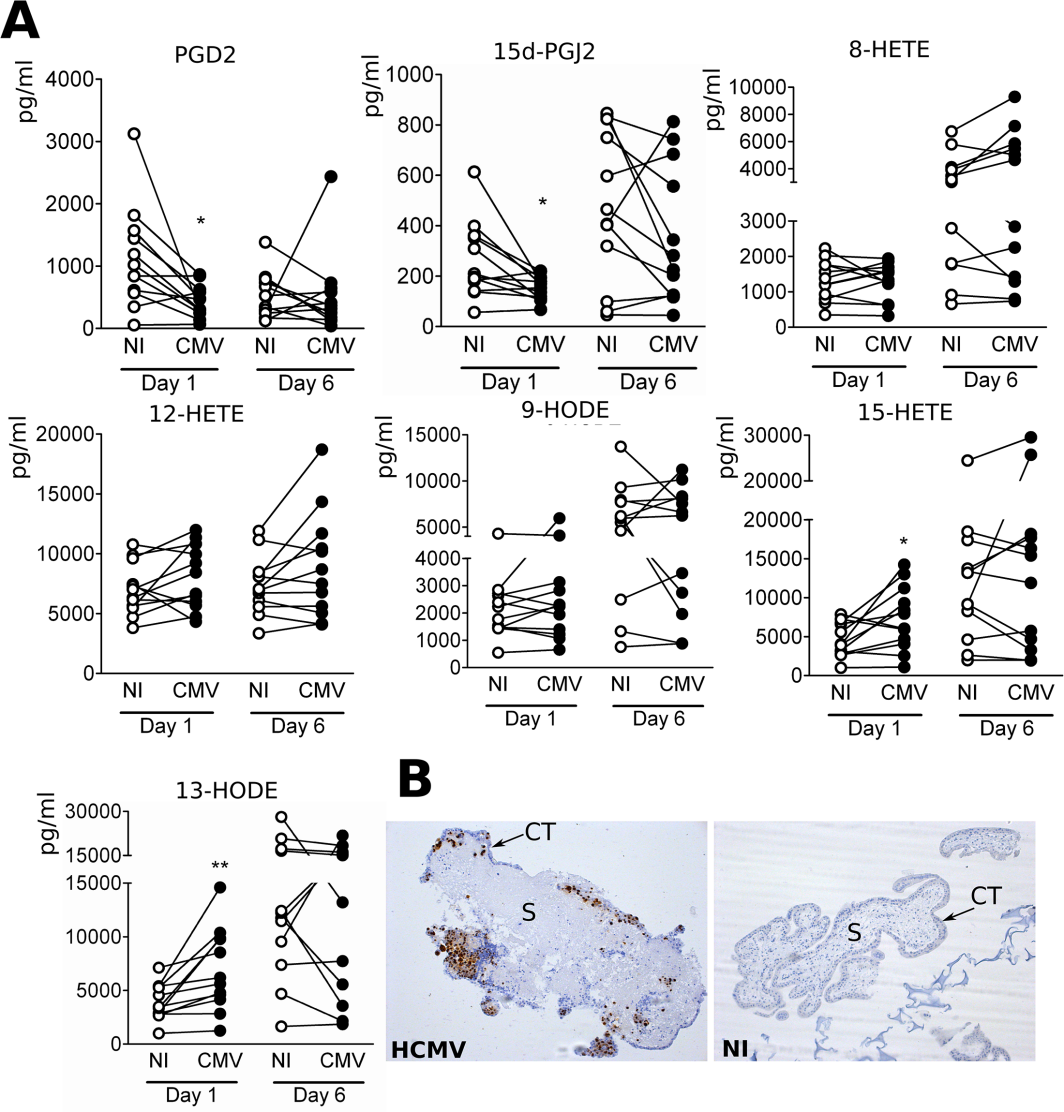
Increased amounts of 15-HETE and 13-HODE secreted by from early placenta explants infected by HCMV. (A) LC-MS/MS analysis of the amounts of PUFA-derived lipids secreted from histocultures from 12 first trimester placentas either infected by HCMV (CMV) or uninfected (NI). *: p < 0.05; **: p < 0.01 (Wilcoxon test). (B) Immunostaining analysis of HCMV antigen IE in infected (HCMV) or uninfected (NI) placental explants cultured ex vivo. Representative views are shown. Note the positive, nuclear IE staining in the cytotrophoblast layer (ST) surrounding the villus stroma (S).

Next, we investigated which PPARγ activator lipids are released upon infection by HCMV using a model closer to the physiological context. To this aim, we used an histoculture model of normal human placental explants that can be infected ex vivo by HCMV and that we described elsewhere [12]. First trimester placenta blocks were obtained from 12 donors and cultured 5 days at the air-liquid interface in the presence of VHL/E-HCMV-producing MRC5 fibroblasts to allow efficient infection. At day 6, placental explants were transferred to MRC5-free culture wells for 1 day or 6 days before culture supernatants were collected for lipid analysis. We used explants from first trimester placenta, since they contain a greater number of invasive extravillous cytotrophoblasts than later stages and because congenital HCMV infection during first trimester confers the most damage to the placenta and/or the fetus. We thus assumed that early placental explants were more likely to recapitulate the situation in vivo. Indeed, we found that only placenta explants growing on supporting infected MRC5 layer could be efficiently infected by HCMV. MRC5 showed no productive infection when the viral inoculum was either UV irradiated or MAFP treated beforehand. 8/12/15-HETE 9/13-HODE, PGD2 and 15d-PGJ_2_ were detectable by LC-MS/MS from 24 hours post installation of the explants in the MRC5-free wells (Fig 3). Although baseline levels were variable among donors, we observed significantly greater amounts of secreted 15-HETE (p< 0.029) and 13-HODE (p< 0.008) in the infectious context when compared to those from the same donor installed on uninfected MRC5, with levels increasing from 1,250 pg/ml (4.2 nM) to 14,600 pg/ml (49 nM)(for 13-HODE) or from 1,007 pg/ml (3 nM) to 14,239 pg/ml (44.5 nM)(for 15-HETE) at day 6 post installation (Fig 3). Statistical analysis confirmed that secreted levels of 13-HODE and 15-HETE were significantly greater in the infected explants as compared to the controls (p< 0.01 and p<0.05, respectively). At day 12, we observed no significant difference in the amounts of 15-HETE or 13-HODE from the histocultures placed in the infectious context as compared as the controls, although their mean values were greater than those at day 1 pi (Fig 3). This suggested that increased amounts at day 12 did not result from increasing number of infected cells within the placenta explants. More importantly, this indicated that secretion of 15-HETE and 13-HODE were rather associated with early stages of infection. In contrast, amounts of PGD2, 15d-PGJ2, 8/12-HETE and 9-HODE were not increased in infected histocultures, at day 6 or 12 (Fig 3A). HCMV infection of the placental explants was checked by immunohistological staining using the antibody specific to IE. Infected explants, but not control explants, showed clear IE immunoreactivity in endothelial, stromal and, mostly, in cytotrophoblasts cells (Fig 3B). Our findings suggest that infection of early placenta results in enhanced secretion of 13-HODE and 15-HETE.

## Discussion

**Fig 4:**
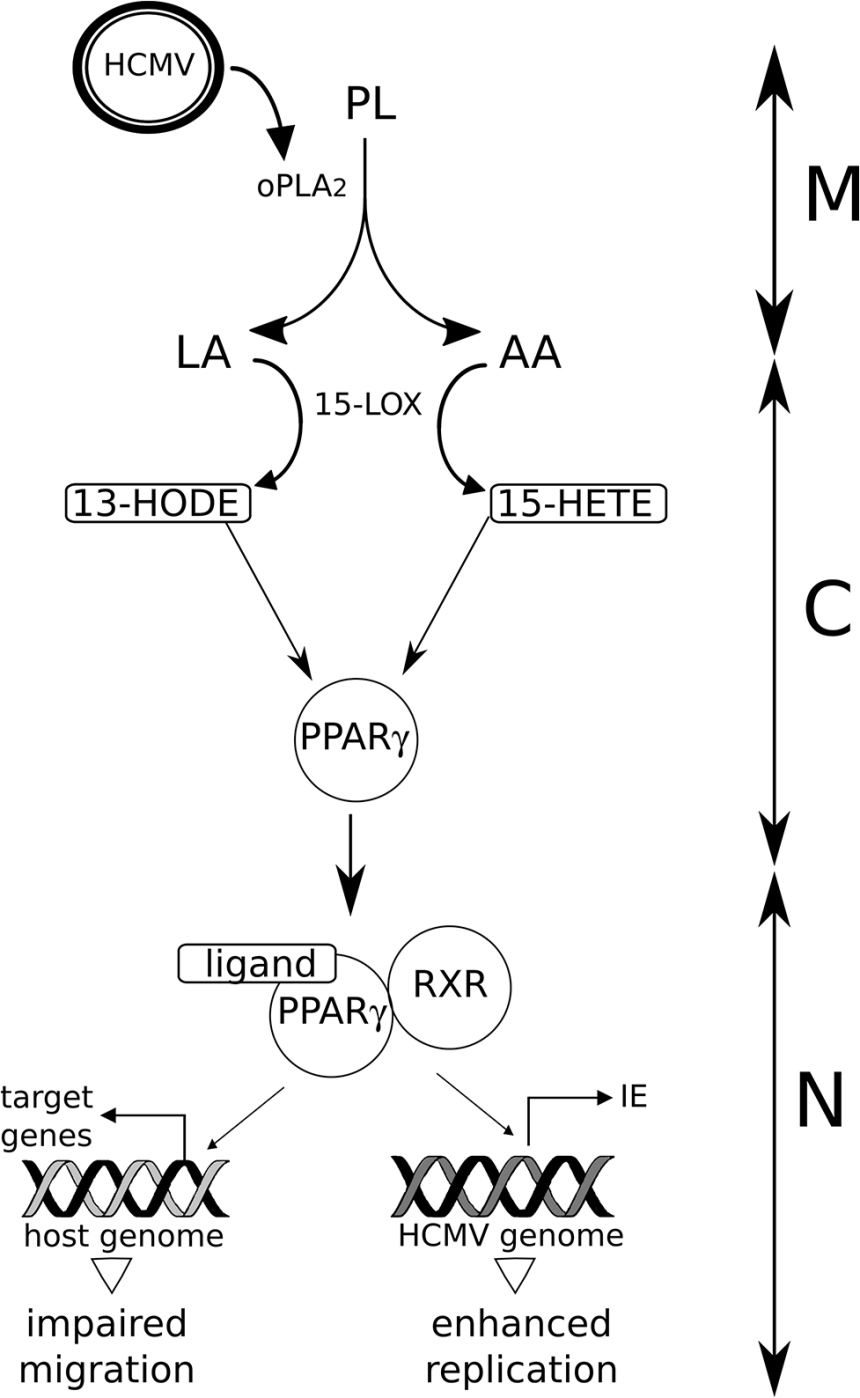
Proposed model of PPARγ activation in HCMV infection of placental cells. HCMV particles (HCMV) carry onboarded cPLA2 (oPLA2) which catalyses linoleic acid (LA) and arachidonic acid (AA) release from host membrane phospholipids (PL). AA and LA undergo oxidization driven by 15-lipoxygenase (15-LOX), which generates 15HETE and 9-HODE, respectively. 15HETE and 9-HODE are activating ligands of PPARγ, which dimerizes with RXR to regulate the expression of the host and virus genomes, resulting in impaired migration abilities in vitro, and enhanced IE transcription and viral replication. M: cell membrane, C: cytoplasm, N: nucleus.

Congenital infection by HCMV is the most frequent congenital infection and accounts for devastating sensorineural and neurodevelopmental sequelae in the severe cases. It also causes placenta dysfunction, which can be associated with pre-eclampsia (PE) like symptoms, IUGR or even stillbirths, and may threaten other organs through the deleterious action of secreted soluble mediators. Given the dramatic health and societal cost of congenital HCMV infection, a better insight on its pathogenesis is needed to provide new therapeutic or prognostic tools. Based on our previous demonstration that PPARγ activation is an early and key effector of HCMV infection in cytotrophoblast cells [8], the goal of this study was to gain novel insight about the modalities of PPARγ activation upon infection. Here, using newly-developed lipidomic approaches, we demonstrate that 15-HETE and 13-HODE are released by HIPEC and placental explants during the early steps of HCMV infection (Fig 4).

In this study, we used two HCMV strains, the laboratory-adapted AD169 and the clinical strain VHL/E. The former harbors a 20-kb genomic deletion which makes the later a more relevant model given that it is used at low passages of amplification. Notably, we found similar results respective to PPARγ activation, HIPEC migration, and onboarded PLA_2_ with AD169 HCMV than what had been reported before with VHL/E HCMV [8, 14]. However, to carry out the LC-MS/MS screening, the experimental procedure and the relatively lower efficiency of producing VHL/E stocks as compared to AD169 prevented us to use low passage VHL/E suspensions. Therefore we elected to use AD169 rather than generate high passage VHL/E for the initial screening in HIPEC, and we used low passage VHL/E for the screening in placental explants. Noteworthy, 13-HODE and 15-HETE were the predominant lipids generated by HIPEC infected by AD169 and by placental explants infected with VHL/E respectively. Also, the results of transwell migration assays, performed with HIPEC infected by VHL/E HCMV, supported those found in wound healing experiments with AD169. Together, these data suggest that AD169 genome alterations do not result in detectable changes respective of HIPEC permissivity and migration abilities, PPARγ activation and 13-HODE and 15-HETE generation.

HIPEC are SV-40 immortalized human EVCT [10]. As such, they probably bear transcriptome or genomic alterations. We failed to generate primary or secondary cultures of EVCT, even from early placentas. Therefore, we elected to investigate the outcomes of infection in placenta explants, a model more relevant to pathophysiology. Noteworthy, the results of the lipid screen using HIPEC and placental explants were similar, showing increased biosynthesis of 13-HODE and 15-HETE in infected explants. However, the limitation of this study is the impossibility to figure out which cell type(s) within the placenta explants actually generate(s) 13-HODE or 15-HETE, or to rule out the possibility of bystander stimulation of 13-HODE / 15-HETE biosynthesis between neighbor cell types within the placental explants.

The amounts of secreted 15-HETE were found at least 2 fold lower than those of cellular 15-HETE. We assumed that this could result from either low levels of secretion or poor stability in the medium. The amounts of both cellular and secreted 13-HODE appeared much greater than those of 15-HETE (> 3 fold), indicating more efficient biosynthesis and/or secretion.

We showed that PLA_2_ activity supplied by the virus (oPLA_2_) is mandatory for 15-HETE and 13-HODE production, consistent with the fact that PLA_2_ drives AA and LA release from membrane phospholipids. We have previously reported that UV-irradiated HCMV could not activate PPARγ in HIPEC [8]. However, we showed here that UV irradiation had no impact on either o PLA_2_ activity, using TLC analysis of HCMV particle lysates (Fig 1A), or on 13-HODE or 15-HETE biosynthesis, by LC-MS/MS analysis using HIPEC infected by UV-irradiated HCMV (Fig 2A). These findings suggest that o PLA_2_ is indeed required, but is not sufficient to trigger PPARγ activation in HIPEC. Viral gene expression seems to be required, possibly through the production of immediate early transcripts that remain to be investigated. These may, in turn, modulate downstream stages of the cascade leading to 15-HETE or 13-HODE biosynthesis.

The present study underscores the role of 15-LOX in HCMV-infected placenta, since 15-LOX is a shared catalytic driver of oxidization of AA or LA that leads to 15-HETE and 13-HODE synthesis. The 15-LOX pathway has been extensively investigated as a key regulator of healthy and pathological vascular system, including vascular plasticity and remodeling; it has been described as critical in the generation as well as the resolution of inflammation; and it has been reported to exert pro-atherosclerotic and anti-atherosclerotic effects [18].

15-HETE was originally identified as a PPARγ agonist in monocytes and macrophages [19, 20], and, as such, could contribute to the anti inflammatory role of the receptor [21]. 15-HETE is associated with PE or vascular pathogeny in a number of studies, and it has been reported to exert important roles in umbilical cord vasoconstriction and vascular remodeling associated with PE [22]. Increased release of 15-HETE in the placenta and in the venous and umbilical cord sera of patients with PE have been reported [23, 24], consistently with increased 15-LOX activity in placenta from PE patients [22]. 15-HETE has been shown to promote HUVEC endothelial cell line proliferation and migration [24]. Here, we found increased release of 15-HETE in histocultures from healthy placenta infected by HCMV ex vivo, outside any context of PE. This finding suggests that 15-HETE secreted in placenta in response to HCMV may have a causative role in PE symptoms associated with congenital HCMV infection. 15-HETE also enhances the expression of placenta growth factor (PLGF) [25]. PLGF is abundantly expressed by trophoblasts and has been proposed to modulate vascular development, stability, and/or function, within the decidua and placental villi [26]. The receptor for PLGF on trophoblast, called fms-like tyrosine kinase (flt-1) receptor, exists as a soluble form, s-flt-1. Strikingly, s-flt-1 levels are increased in the amniotic fluid of patients with congenital HCMV infection [27], and could thereby represent a negative modulation of excessive PLGF signal due to increased levels of 15-HETE resulting from HCMV infection. Besides, 15-HETE appeared to be also a negative regulator of 15-LOX isoform 2 gene expression through an autocrine feedback loop involving binding onto the 15-LOX-2 gene promoter of PPARγ activated by 15-HETE [28]. Lastly, 15-HETE is an endogenous regulator of prostacyclin, a prostanoid which is a key regulator of vascular homeostasis during pregnancy in particular [29].

13-HODE was originally identified as a strong PPARγ ligand in the monkey kidney cell line CV1 and in human macrophages [20]. Hence, similar to 15-HETE, 13-HODE contributes to the anti-inflammatory role of the receptor [21]. In human trophoblasts, 13-HODE was previously reported to increase human chorionic gonadotropin production but showed no effect on the differentiation markers syncitin, cyclin E or p27 [17]. In addition to linoleic acid, oxidized low-density lipoproteins (oxLDL) are a natural source of 13-HODE [30–32]. 13-HODE might play a role in the inhibition of trophoblast invasion mediated by oxidized LDLs [33]. 13-HODE can also modulate the immunological function of oxLDL: notably, the capacity of oxLDL to generate activating dendritic cells from differentiating monocytes was shown to be inhibited by the presence of 13-HODE [34]. 13-HODE is also an activating ligand of the transient receptor potential vanilloid type 1 (TRPV1), a non-selective cation channel with a wide distribution throughout the central and peripheral nervous systems [35, 36]. TRPV1 integrates painful stimuli, though the role of 13-HODE as such a mediator of inflammatory pain responses remains unclear [37]. It has been proposed that PPARγ may be involved in the pathophysiology of IUGR and/or PE [38]. In PE, lipid deposition in maternal spiral arteries, namely acute atherosis, resembles early stages of atherosclerosis [39]. Yet, 13-HODE shows atherogenic activity and LDLs are abundant in atherosclerotic lesions [30]. Therefore, it is tempting to speculate that 13-HODE production might have a role in the onset of PE-like symptoms.

In conclusion, we reveal that HCMV-infected HIPEC cytotrophoblasts and placental explants release predominantly increased amounts of 15-HETE and 13-HODE, two PUFA-derived mediators that lie at the crossroads of a number of pathways modulating inflammation as well as vasculature, during pregnancy.

## Supporting Information

S1 Fig UV irradiation of HCMV particles does not prevent infection but abolishes expression of the viral genome. Shown are representative immunofluorescence analyses of the input HCMV tegument protein pp65 (pp65), performed 30 min post infection (pi) and of the HCMV Immediate Early antigen (IE), performed 24 hours pi, in HIPEC infected by live HCMV (live) or HCMV irradiated by UV light (UV), at a MOI of 3, or uninfected HIPEC (NI). Scale bar: 25 μm.
(TIFF)
