# Supplementary figures and images for: Cytomegalovirus Infection Triggers the Secretion of the PPARγ Agonists 15-Hydroxyeicosatetraenoic Acid (15-HETE) and 13-Hydroxyoctadecadienoic Acid (13-HODE) in Human Cytotrophoblasts and Placental Cultures
Source: PLoS One. 2015 Jul 14;10(7):e0132627. doi: 10.1371/journal.pone.0132627 (PMC4501751; doi:10.1371/journal.pone.0132627)

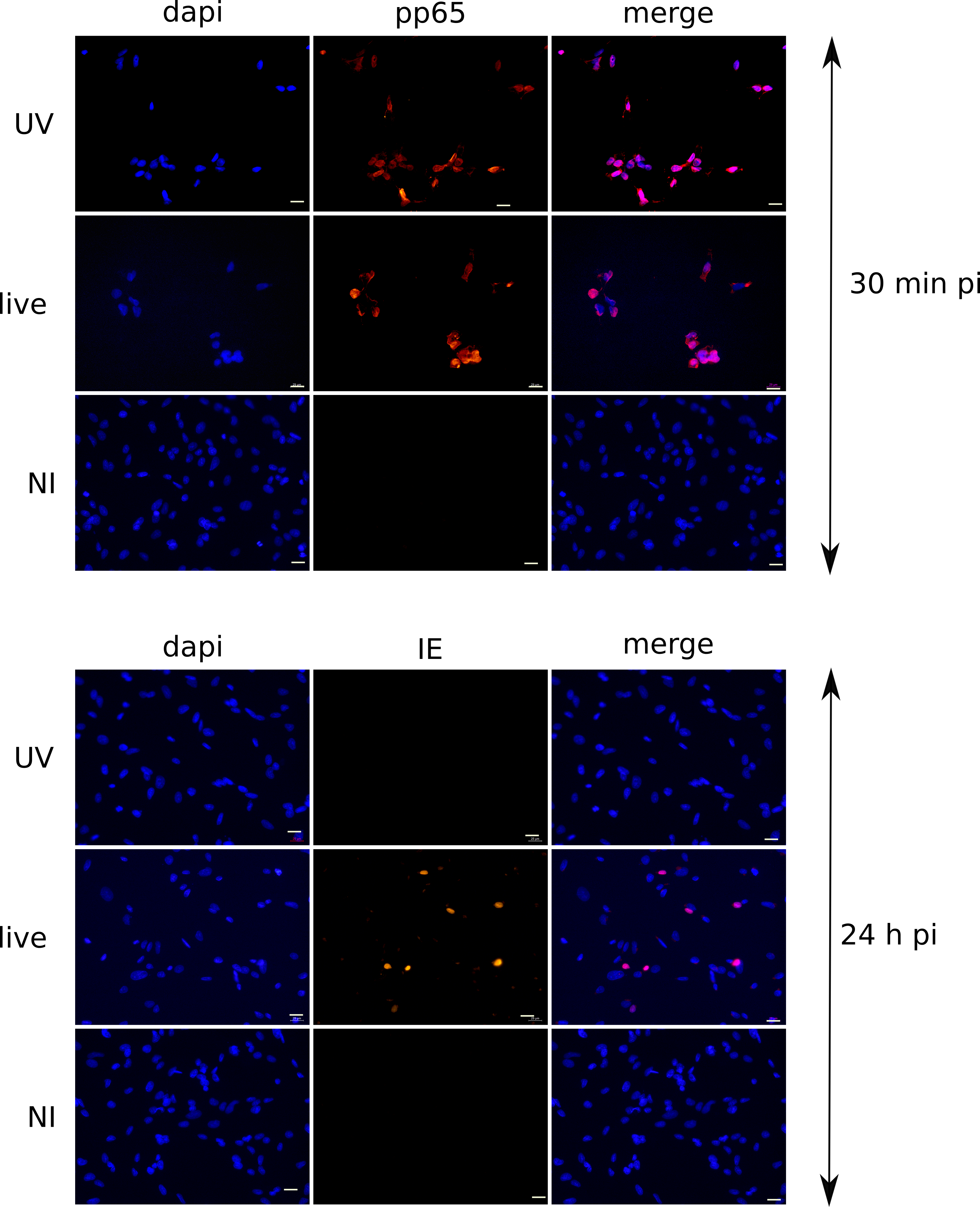

Supplement: S1 Fig — Shown are representative immunofluorescence analyses of the input HCMV tegument protein pp65 (pp65), performed 30 min post infection (pi) and of the HCMV Immediate Early antigen (IE), performed 24 hours pi, in HIPEC infected by live HCMV (live) or HCMV irradiated by UV light (UV), at a MOI of 3, or uninfected HIPEC (NI). Scale bar: 25 μm. (TIFF) [file pone.0132627.s001.tiff]
